# Diversity of clinical, radiographic and genealogical findings in 41 families with amelogenesis imperfecta

**DOI:** 10.1590/1678-7757-2018-0359

**Published:** 2019-04-01

**Authors:** Daniela Adorno-Farias, Ana Ortega-Pinto, Paulina Gajardo, Ana Salazar, Irene Morales-Bozo, Fabiola Werlinger, Sandra Rojas-Flores, Alfredo Molina-Berríos, Sonia Echeverría-López, José Jara-Sandoval, Lilian Jara, Blanca Urzúa

**Affiliations:** 1 Universidad de Chile, Facultad de Odontología, Departamento de Patología y Medicina Oral, Santiago, Chile.; 2 Universidad de Chile, Facultad de Odontología, Santiago, Chile.; 3 Universidad de Chile, Facultad de Odontología, Programa de Magister en Ciencias Odontológicas, Santiago, Chile.; 4 Universidad de Chile, Facultad de Odontología, Instituto de Investigación en Ciencias Odontológicas, Santiago, Chile.; 5 Universidad de Chile, Facultad de Odontología, Departamento del Niño y Ortopedia Dentomaxilar, Santiago, Chile.; 6 Universidad de Chile, Facultad de Medicina, Instituto de Ciencias Biomédicas, Santiago, Chile.; 7 U-Odontología: Red de Investigación en Enfermedades Orales Complejas, Santiago, Chile.

**Keywords:** Amelogenesis Imperfecta, Dental enamel, Malformations, Hypoplasia, Hypomineralization

## Abstract

Amelogenesis imperfecta (AI) is a group of enamel development disorders that alter the structure and chemical composition of the tissue. There is great variability in the clinical presentation; according to Witkop, AI can be categorized into 14 subtypes, which makes its diagnosis extremely complex.

**Objective:**

This study aimed to describe and determine the frequency of clinical and radiographic features and inheritance patterns found in 41 Chilean families diagnosed with diverse types of AI.

**Material and Methods:**

We analyzed the clinical records, photographs, pedigrees and radiographs of 121 individuals recruited between 2003 and 2016. All of the information was included in a database that was analyzed using the application Stata 14.

**Results:**

The 72 affected individuals had average age of 16 years, and no sex association with the presence of AI was found. The most frequent clinical subtypes were as follows: 43% hypomature, 25% hypoplastic, 21% hypomature/hypoplastic, 7% hypocalcified and 4% hypocalcified/hypoplastic. The number of severely affected teeth was 22, which occurred in the patients with hypocalcified and hypocalcified/hypoplasic AI who presented the highest number of damaged teeth. Caries and periodontal disease were found in 47 and 32% of the patients, respectively. Malocclusions were observed in 43% of the individuals with AI, with open bite being the most frequent. Radiographically, the thickness of the enamel decreased in 51% of the patients, and 80% showed decreased radiopacity of the enamel compared to that of dentin. Autosomal dominant inheritance pattern was found in 37% of the families with hypoplastic AI, and autosomal recessive pattern was present in 56% of the other clinical subtypes, but more frequently in those affected with hypomature and hypocalcified AI.

**Conclusion:**

Of the five clinical subtypes, autosomal recessive hypomature, autosomal dominant hypoplastic and autosomal recessive hypomature/hypoplastic AI were the most prevalent subtypes in this group.

## Introduction

Normal enamel is synthesized during tooth development as an extracellular matrix in a process called amelogenesis, which occurs in two stages. In the secretory stage, the ameloblast produces a partially mineralized protein matrix, which will correspond to the adult enamel. In the maturation stage, the protein matrix is degraded, and mineralization is completed.^1^ ^,^ ^2^

Amelogenesis imperfecta (AI) is a group of low-prevalence hereditary conditions that cause alterations in the structure and chemical composition of the enamel matrix during development.^1^ ^-^ ^3^ Currently, the diagnosis of AI involves a clinical and radiographic examination, and when possible, morphological analysis, using ground sections and scanning electron microscopy of the teeth, and molecular genetic analysis of the DNA samples can be performed.^3^ ^-^ ^5^

AI is classified into three main types that are related to the stages of the tissue formation process.^6^ A fault in the secretory stage of amelogenesis produces the hypoplastic type of AI, which is characterized by enamel that is thinner than normal and that contrasts normally from dentine in the radiographic analysis.^7^ ^,^ ^8^ In hypocalcified AI, there is an alteration in the initial mineralization of the secretory stage; the enamel initially develops normal thickness, is orange-yellow at eruption and consists of poorly calcified matrix that is rapidly lost during normal function. In addition, the enamel has a lower radiopacity than the dentin.^6^ ^,^ ^7^ In hypomature AI, the defect occurs in the maturation stage of the enamel; is of normal thickness but has a mottled appearance; is slightly softer than normal enamel; and chips from the crown. Radiographically, it presents with approximately the same radiodensity as that of dentin.^6^ ^,^ ^9^

To adequately evaluate AI, a differential diagnosis with other defects and an investigation to determine if the enamel alterations are linked to environmental disturbances or have a discernible genetic transmission pattern are necessary.^2^ ^,^ ^10^ ^,^ ^11^ The signs frequently found in the enamel are a decrease in thickness, rough texture, mottled appearance, the presence of cavitations (pits), horizontal or vertical bands and a dull white, yellow or brown color. In addition, a delay in eruption, dental impaction, anterior or lateral open bite, ogival palate, gingival inflammation, teeth with a decreased coronary sector and multiple diastemas can be observed.^12^ ^-^ ^14^

Because AI is a rare and heterogeneous condition from a clinical and genetic point of view, dentists, in general, have difficulty in making a correct diagnosis regarding the presence of AI and the determination of its clinical subtype. In our experience, many professionals who do not know about this condition and its consequences in long-term treatment choose to refer these patients, which contributes to a greater psychosocial impact on them because they feel marginalized and are often left without a specific treatment solution and prognosis of their condition.

Given the above factors, the literature reports few AI case series that systematically describe the characteristics associated with this phenotype. Moreover, in our country, there have been no studies that determine the prevalence, the different subtypes of AI, or the patterns of inheritance associated with each case. In the absence of previous data to refute this, our null hypothesis proposes that the clinical, radiographic and genealogical findings observed in the group of Latin American patients studied are similar to those reported in other populations. Therefore, this study aims to describe and determine the frequency of clinical and radiographic features and inheritance patterns found in 41 Chilean families diagnosed with AI and, thus, contribute to the identification of the clinical diagnostic tools used during routine dental practice.

## Material and methods

This research was conducted under the framework of a funded research project, which has the authorization of the Ethics Committee and the Institutional Biosafety Committee of the Faculty of Dentistry and the University of Chile (Certificate No. 2013/06 and FDO No. 224 of June 2013, respectively), and in full accordance with the ethical principles of the Declaration of Helsinki and local regulations.

One hundred twenty-one individuals belonging to 41 Chilean families with AI were recruited between 2003 and 2016 and included in this study. Each of the patients was evaluated by two oral pathologists. A record of personal and family history was completed including possible alterations during the intrauterine development stage. Affected and unaffected individuals were evaluated clinically for the presence of skin, hair, fingernail, kidney, cognitive, gingival, visual, auditory and osseous abnormalities known to be a sign of systemic or syndromic conditions that could be associated with AI. In-depth interviews were conducted with at least two family members by a geneticist in order to construct their genealogies and analyze the inheritance pattern. Additionally, clinical photographs were taken, and complementary tests were requested such as periapical and panoramic radiographs.

The presence of alterations in the enamel was recorded in relation to the glossiness, texture, color, thickness and surface defects. The gloss of the enamel was considered abnormal when it had an opaque appearance. The texture was evaluated by gently passing the dental probe over the enamel, and the thickness of the enamel was considered diminished when there was presence of generalized diastemas, pits or bands; the evaluation was complemented by the radiographic image. A radiographic analysis was conducted to evaluate the radiopacity and thickness of the enamel as well as the presence of dental and bone alterations.

The diagnosis of AI and its respective subtypes was based on the criteria described in the classification of Witkop^6^ (1989); however, we considered only the following three main types of AI and their possible combinations: “type I-Hypoplastic, type II-Hypomaturation and type III-Hypocalcified”. Taurodontism was recorded as one more characteristic, among several others, that are associated with AI and not as “type IV-Hypomaturation-hypoplastic with taurodontism” from the Witkop's classification.

All of the information collected was included in a single database, and the statistical analysis was conducted using the Stata computer program, version 4 (StataCorp, College Station, TX, USA). The continuous variables were assessed for normality using the Shapiro-Wilk test. The data are presented as either the mean and standard deviation or as the median and range. The data were compared using the Fisher's exact, Wilcoxon or Kruskal-Wallis test. The significance was determined at a value of P<0.05.

## Results

### Sample description

A total of 121 individuals were evaluated from 41 Chilean families with an AI diagnosis; 58% of the cases were female (Table 1). Most of the evaluated patients presented clinically with AI (60%), and the remaining individuals were direct relatives who did not manifest the disease. Although the number of women was higher in the group of affected individuals, no sex association was found in relation to the presence of AI (*P*=0.320). The group of individuals affected with AI had a significantly lower median age than the unaffected group (*P*=0.0004) (Table 1).

**Table 1:** Distribution of individuals affected and not affected with Amelogenesis Imperfecta from 41 Chilean families, according to sex and age

		FEMALE		MALE		TOTAL
CONDITION	No. (%)	Med Age (Rank)	No. (%)	Med Age (Rank)	No. (%)	Med Age (Rank)
Non affected^v^	31 (44)	37 (2-64)	18 (35)	19 (1-63)	49 (40)	37 (1-64)^a^
Affected by AI&	39 (56)	13 (2-44)	33 (65)	17 (6-84)	72 (60)	16 (2-84)^a^
TOTAL	70 (100)	28 (2-64)	51 (100)	17 (1-84)	121 (100)	21 (1-84)

Data are presented as No. (%) and Median (yr) (Rank).

v 1 non-affected woman without age data,

& 2 affected woman without data od age.

a Wilcoxon test, P=0.0004

### Genealogical analysis

A significantly higher number of families affected with AI (56%) presented an autosomal recessive inheritance pattern (*P*=0.006) (Table 2), followed by 15 families that showed an autosomal dominant inheritance pattern, which represented 37% of the sample.

**Table 2:** Inheritance patterns for the 41 Chilean families with Amelogenesis Imperfecta according to the subtype of AI diagnosed

INHERITANCE PATTERN	HPAI		HMAI		HCAI		HM/HPAI		HC/HPAI		TOTAL	
	No.	%	No.	%	No.	%	No.	%	No.	%	No.	%
Autosomal Dominant	5	83	5	26	1	20	2	25	2	67	15	37
Autosomal Recessive	0	0	14	74	4	80	4	50	1	33	23^a^	56
X-linked	0	0	0	0	0	0	0	0	0	0	0	0
Two possible patterns*	0	0	0	0	0	0	2	25	0	0	2	5
Undetermined	1	17	0	0	0	0	0	0	0	0	1	2
TOTAL families	6	100	19	100	5	100	8	100	3	100	41	100

* Autosomal Recesive or Esporadic/Autosomal Dominant or X-linked;

a Fisher exact test P=0.006

### Intraoral clinical characteristics

A total of 93% of the individuals had normal-appearing mucosa (Table 3), and 7% had alterations of the mucosa that corresponded to traumatic and/or reactional injuries, canker sore and hyperpigmentation of the gum. Regarding the type of dentition, 71% of the individuals had permanent dentition with a median of 27 permanent teeth (range 0-32) present in the mouth at the time of the examination. Caries were observed in 47% of the patients, and restorations were observed in 67% of the patients. A total of 39% of the patients had undergone exodontias, and the median number of teeth clinically observed that were severely affected with AI in the sample of patients was 22 (range 4-31) (Table 3).

**Table 3:** Distribution of the intraoral clinical characteristics observed in the individuals affected with Amelogenesis Imperfecta according to clinical subtype

INTRAORAL CLINICAL CHARACTERISTICS		HPAI	HMAI	HCAI	HM/HPAI	HC/HPAI	TOTAL
		n=18	n=31	n=5	n=15	n=3	n=72
		No. (%)	No. (%)	No. (%)	No. (%)	No. (%)	No. (%)
MOUTH							
Normal mucosa		16 (89)	30 (97)	5 (100)	13 (87)	3 (100)	67 (93)
Altered Mucosa		2 (11)	1 (3)	0 (0)	2 (12)	0 (0)	5 (7)
Low/high lip frenum insertion		5 (28)	6 (19)	2 (40)	5 (33)	1 (33)	19 (26)
Short lip frenum		0 (0)	3 (10)	0 (0)	1 (7)	0 (0)	4 (6)
DENTITION							
Permanent Dentition		16 (89)	23 (74)	4 (80)	5 (33)	3 (100)	51 (71)
Mixed Dentition		2 (11)	8 (26)	1 (20)	8 (53)	0 (0)	19 (26)
Primary Dentition		0 (0)	0 (0)	0 (0)	2 (13)	0 (0)	2 (3)
Diastemas		10 (56)	4 (13)	1 (20)	5 (33)	3 (100)	23 (32)
TREATMENTS							
Restorations		14 (78)	21 (68)	2 (40)	9 (60)	3 (100)	48 (67)
Veneers or fixed prostheses		5 (28)	4 (13)	2 (40)	1 (7)	1 (33)	13 (18)
Exodontias		10 (56)	13 (42)	2 (40)	2 (13)	1 (33)	28 (39)
CONDITIONS / PATHOLOGIES							
Caries		7 (39)	15 (48)	3 (60)	7 (47)	2 (67)	34 (47)
Agenesis		0 (0)	3 (10)	1 (20)	2 (13)	1 (33)	7 (10)
Eruption delay		0 (0)	1 (3)	1 (20)	1 (7)	0 (0)	3 (4)
Periodontal disease		5 (28)	9 (29)	3 (60)	6 (40)	0 (0)	23 (32)
Gingival hyperplasia		1 (6)	2 (6)	0 (0)	0 (0)	0 (0)	3 (4)
Ogival Palate		3 (17)	7 (23)	2 (40)	3 (20)	1 (33)	16 (22)
OCCLUSSION							
Malocclusions (total):		9 (50)	9 (29)	4 (80)	8 (53)	1 (33)	31 (43)
	Anterior Openbite	5 (28)	2 (6)	2 (40)	2 (13)	1 (33)	12 (17)
	Lateral bite	1 (6)	2 (6)	1 (20)	1 (7)	0 (0)	5 (7)
	Crossbite	2 (11)	2 (6)	0 (0)	3 (20)	0 (0)	7 (10)
	Inverted bite	1 (6)	1 (3)	1(20)	1 (7)	0 (0)	4 (6)
	Bis to bis bite	0 (0)	2 (6)	0 (0)	1 (7)	0 (0)	3 (4)
SENSITIVITY							
Sensitivity associated to (total):		6 (33)	6 (19)	2(40)	2 (13)	3 (100)	19 (26)
	Thermal changes	2 (11)	2 (6)	0 (0)	1 (7)	3 (100)	8 (11)
	Chemical changes	1 (6)	3 (10)	0 (0)	0 (0)	0 (0)	4 (6)
Both (thermal and Chemical)		3 (17)	0 (0)	2 (40)	1 (7)	0 (0)	6 (8)
OTHERS							
Other alterations *		6 (33)	16 (52)	1 (20)	8 (53)	1 (33)	32 (44)

AIHP=AI hypoplastic; AIHM=AI hypomature; AIHC=AI hypocalcified; AI HM/HP=AI hypomature/hypoplastic; AIHC/HP=AI hypocalcified/hypoplastic; GS=Salivary Glands.

* Fibromatosis, anterior covering bite, increased overjet, dental invaginations, irritative fibroma, Fordyce condition, angular cheilitis, bruxism, erythematous plaques on tongue, semi-bifid tongue, fissured tongue, microdontia, teeth with shape alteration, macrodontia, teeth included, extrinsic dental staining, change in coloration by tetracycline (each with 1%), in addition to crowding, prognathism, overbite, palatine torus, carabelli tubercle and pits and fissures in teeth, each with 3% and geographical tongue with 4%

Malocclusions were observed in 43% of the individuals with AI, with open bite being the most frequent form (17%). On the other hand, 26% of the patients presented some type of dental symptomatology, and sensitivity to thermal changes was the most frequently reported symptom (11%). It is interesting to note that periodontal disease, the presence of diastemas, high or low lip frenum insertion and ogival palate occurred with frequencies equal or greater than 22% (Table 3).

### General and specific clinical characteristics of enamel

With regard to glossiness and texture, 41% of the patients showed an opaque enamel, and 46% of them showed that the texture of the tissue was rough (Table 4). The most commonly observed enamel coloration (65% of the patients) was white/opaque with absence of translucency, which was more frequent than other colorations (P<0.0001). Opaque white spots were observed in a high percentage of the patients with AI (77%). Regarding surface defects of the enamel, 29% of the individuals had pitting and 65% showed wear (Table 4).

**Table 4:** Distribution of the general and specific clinical characteristics of the enamel observed in the individuals affected with Amelogenesis Imperfecta (AI) according to clinical subtype

CLINICAL CHARACTERISTICS OF ENAMEL	HPAI	HMAI	HCAI	HM/HPAI	HC/HPAI	TOTAL
	n=18	n=31	n=5	n=15	n=3	n=72
	No. (%)	No. (%)	No. (%)	No. (%)	No. (%)	No. (%)
GLOSSINESS&						
Normal	10 (59)	20 (69)	0 (0)	11 (73)	0 (0)	41 (59)
Opaque	7 (41)	9 (31)	5 (100)	4 (27)	3 (100)	28 (41)
TEXTURE&						
Normal	9 (53)	19 (66)	0 (0)	9 (60)	0 (0)	37 (54)
Rough	8 (47)	10 (34)	5 (100)	6 (40)	3 (100)	32 (46)
COLOR&						
White/opaque	12 (71)	22 (76)	0 (0)	11 (73)	0 (0)	45 (65)^a^
Yellow	5 (29)	5 (17)	2 (40)	4 (27)	1 (33)	17 (25)
Brown	0 (0)	0 (0)	3 (60)	0 (0)	1 (33)	4 (6)
White-yellow	0 (0)	1 (3.5)	0 (0)	0 (0)	1 (33)	2 (3)
Yellow-brown	0 (0)	1 (3.5)	0 (0)	0 (0)	0 (0)	1 (1)
THICKNESS&						
Normal	3 (18)	26 (90)^b^	0 (0)	9 (60)	0 (0)	38 (55)
Decreased	14 (82)	3 (10)	5 (100)	6 (40)	3 (100)	31 (45)
LINE BANDING						
Horizontal	5 (29)	3 (10)	0 (0)	0 (0)	0 (0)	8 (12)
Vertical	2 (12)	3 (10)	0 (0)	3 (20)	0 (0)	8 (12)
SPOTTING						
White/opaque spots	9 (53)	29 (100)	1 (20)	14 (93)	0 (0)	53 (77)
Yellow-brown spots	4 (24)	8 (28)	1 (20)	5 (33)	1 (33)	19 (28)
SURFACE DEFECTS						
Pits or cavitation	7 (41)	6 (21)	2 (40)	4 (27)	1 (33)	20 (29)
Wear	16 (94)	13 (45)	4 (80)	10 (67)	2 (67)	45 (65)
WITHOUT DATA&						
No. Patients	1 (6)*	2 (7)	0 (0)	0 (0)	0 (0)	3 (4)

& Patients without data in these characteristics,

* all teeth restored at the time of the examination.

a, b Fisher's exact test P<0.0001

### Radiographic characteristics of the enamel

Radiographic analysis (Table 5) showed that normal and decreased enamel thickness occurred equally in individuals with AI. A decrease in the radiopacity of the enamel compared to that of dentine was observed in 80% of the patients. In this sample, only 16% of the patients presented taurodontism.

**Table 5:** Distribution of the radiographic characteristics observed in the individuals affected with Amelogenesis Imperfecta according to clinical subtype

RADIOGRAPHIC CHARACTERISTICS OF ENAMEL	HPAI	HMAI	HCAI	HM/HPAI	HC/HPAI	TOTAL
	n=18	n=31	n=5	n=15	n=3	n=72
	No. (%)	No. (%)	No. (%)	No. (%)	No. (%)	No. (%)
THICKNESS						
Normal	1 (9)	19 (79)	1 (20)	6 (50)	0 (0)	27 (49)
Decreased	10 (91)	5 (21)	4 (80)	6 (50)	3 (100)	28 (51)
RADIOPACITY*						
Normal	2 (18)	6 (25)	0 (0)	3 (25)	0 (0)	11 (20)
Decreased	9 (82)	18 (75)	5 (100)	9 (75)	3 (100)	44 (80)
PRESENCE OF						
Taurodontism	0 (0)	1 (4)	2 (40)	5 (42)	1 (33)	9 (16)
WITHOUT DATA						
No. Patients	7 (64)	7 (29)	0 (0)	3 (25)	0 (0)	17 (31)

* Radiopacity of the enamel in relation to the dentin

### Clinical analysis of phenotypes

**Figure 1:**
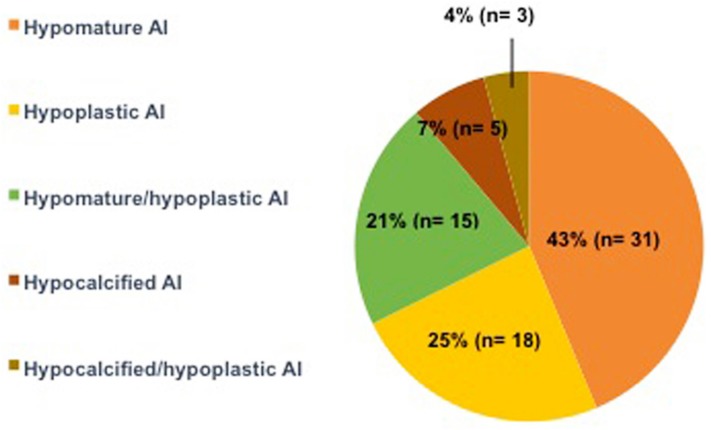
Pie chart showing the distribution of clinical subtypes in the 72 patients affected with Amelogenesis Imperfecta

Five clinical subtypes of AI were identified (Figure 1). The most frequently observed clinical subtypes in the 72 affected patients were hypomature AI (43%), followed by hypoplastic AI (25%) and hypomature/hypoplastic AI (21%). Clinical subtypes of hypocalcified AI and hypocalcified/hypoplastic AI were found in 7% and 4% of the patients, respectively.

#### Hypoplastic Amelogenesis Imperfecta (HPAI)

**Figure 2:**
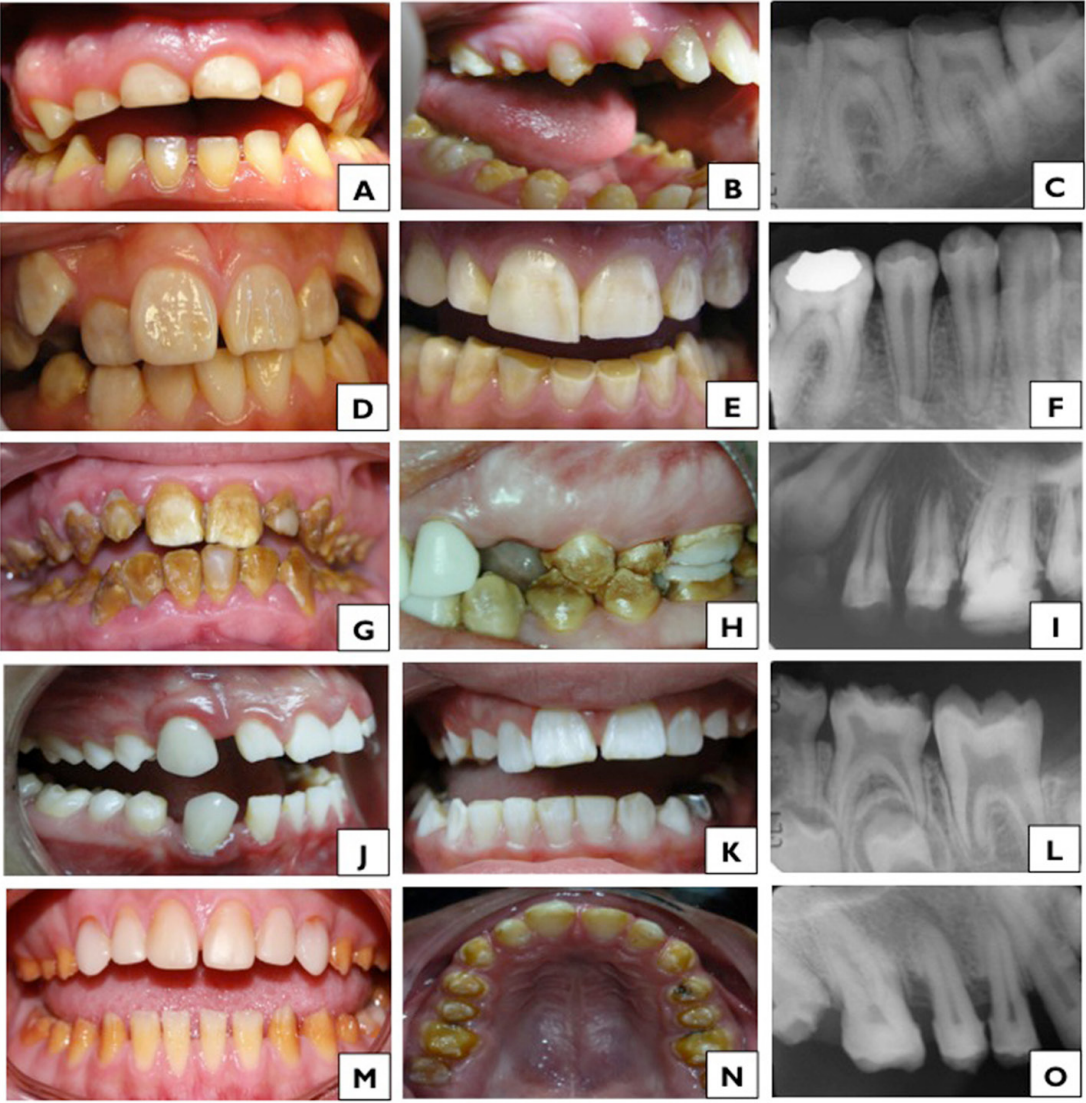
Clinical subtypes of Amelogenesis Imperfecta found in this study; A, B, C: Clinical subtype of Hypoplastic Amelogenesis Imperfecta; D, E, F: Subtype of Hypomature AI; G, H, I: Subtype of Hypocalcified AI; J, K, L: Subtype of Hypomature-hypoplastic AI; M, N, O: Subtype of Hypocalcified-hypoplastic AI

The 18 cases of HPAI presented enamel thickness alterations such as a generalized decrease in thickness, which occurred in 82% of the cases (Table 4, Figures 2A and 2B), cavitations or pits in 41% of the cases, and the presence of horizontal bands in 29% of the patients (Table 4). Changes in enamel thickness were not similar within the same family. Different degrees of affection were also observed in the same individual as shown in Figure 2B. Diastemas were observed in 56% of cases, and dentin sensitivity was observed in 33% of the patients (Table 3). Dental wear and exodontias were very frequent in this group, which were observed in 94% and 56% of the cases, respectively (Tables 3 and 4, Figures 2A and 2B). Other clinical alterations were opaque white spots in 53% of the patients, rough texture in 47% of the patients, and opaque enamel in 41% of cases (Table 4).

Alterations in occlusion were observed in 50% of the patients, with open bite being the most frequent finding (28% of the cases) (Table 3). Of the 18 cases of HPAI, radiographic images were available in 11 cases with evident enamel thickness reduction in 91%, and radiopacity in 82% of the cases (Table 5 and Figure 2C). Of the 11 cases analyzed, none presented taurodontism (Table 5). Of the 6 families with HPAI, 83% presented an autosomal dominant pattern of inheritance (Table 2).

#### Hypomature Amelogenesis Imperfecta (HMAI)

Thirty-one cases were classified in this group, from which the most frequently observed enamel characteristics were opaque white spots in 100% of the cases, normal enamel thickness in 90% of the cases (P<0.0001), white/opaque enamel color in 76% of the cases, enamel with normal glossiness in 69% of the cases and normal texture in 66% of the cases (Table 4 and Figures 2D and 2E). It is important to highlight that periodontal disease and malocclusions affected 29% of the patients (Table 3). In the radiographic study, a normal enamel thickness was observed in 79% of the cases, and a decrease in radiopacity was observed in 18 cases (75%) (Table 5, Figure 2F). In this subtype of AI, the most observed pattern of inheritance was the autosomal recessive type (14 families, 74%) (Table 2).

#### Hypocalcified Amelogenesis Imperfecta (HCAI)

The 5 cases of HCAI had opaque and rough enamel and decreased thickness (Table 4). In 60% of the cases, the enamel was brown, and in the remaining two cases, it was yellow (Table 4, Figures 2G and 2H). Regarding other clinical characteristics, 80% of the cases presented malocclusions, which were mainly open bite, whereas 60% of the cases presented periodontal disease, and 40% presented sensitivity to both thermal and chemical stimuli (Table 3). This clinical subtype presented more affected teeth than the other clinical subtypes. The imaging showed that all of the cases had less radiopacity of the enamel compared to dentin (Figure 2I), and 80% of the cases presented a decrease in the enamel thickness of the erupted teeth (Table 5). An interesting finding was that 40% of the patients presented taurodontism (Table 5). In this group, 80% of the families presented an autosomal recessive inheritance pattern (Table 2).

#### Hypomature/Hypoplastic Amelogenesis Imperfecta (HM/HPAI)

In this group, both HMAI and HPAI characteristics were observed in the same patient (Figures 2J and 2K). The characteristics of HMAI were as follows: opaque enamel in 27% of cases, rough enamel in 40% of the patients, white/opaque enamel in 73% of cases and opaque white spots in 93% of cases (Table 4). The following characteristics of HPAI were observed: uniformly reduced thickness in 40% of cases, vertical lines in 20% and pits in 27% of cases (Table 4). Among the intraoral alterations, periodontal disease was observed in 40% of cases, malocclusions in 53% and the presence of diastemas in 33% of cases (Table 3). The radiographic study showed a decrease in radiopacity in 75% of cases (Table 5 and Figure 2L), a generalized decrease in enamel thickness in 50% of cases, and taurodontism in 42% of cases (Table 5). In this subtype of AI, 50% of the families showed an autosomal recessive inheritance pattern (Table 2).

#### Hypocalcified/Hypoplastic Amelogenesis Imperfecta (HC/HPAI)

This group was formed by 3 individuals with presence of restorations, sensitivity to thermal changes and diastemas (Table 3). These 3 patients presented rough opaque enamel and a generalized decrease in thickness (Table 4, Figures 2M and 2N). The color of the enamel varied from brown to yellow and white-yellow, and 1 patient presented with pits or cavitations (Table 4). The radiographic study showed a generalized decrease in enamel thickness and decreased radiopacity compared to dentin (Table 5, Figure 2O). Of the 3 families analyzed, only 2 had autosomal dominant inheritance (Table 2).

## Discussion

AI is a term that encompasses several hereditary conditions that affect the structure and appearance of tooth enamel.^2^ ^,^ ^6^ ^,^ ^8^ The lack of local information and the information in the literature about the clinical and radiographic signs associated with different subtypes of AI was the motivation for the development of this study, in which 121 individuals belonging to 41 Chilean families were evaluated from a clinical, radiographic and genealogical point of view.

### Sample and clinical analysis

The patients were mainly young, with a median age of 16 years (range 2-84 years). There were similar frequencies of men and women, which coincide with results reported in the literature for the profile of patients with AI.^2^ ^,^ ^6^ ^,^ ^11^ Although hypoplastic AI has been described as the most prevalent subtype occurring in 43.7% of the cases,^15^ ^,^ ^16^ the most frequent type found in this work was hypomature AI followed by hypoplastic AI. In addition, the third most frequent subtype observed in patients consisted of mixed characteristics of hypomature and hypoplastic AI.

Even though an association between AI and several extraoral alterations has been described in the literature^18^ ^-^ ^21^, most of the individuals in this study were systemically healthy and were not receiving pharmacological treatment at the time of the examination, which supports the diagnosis of AI and excludes a possible environmental cause due to the ingestion of medications.

The majority of individuals with AI had exclusively permanent dentition and a higher frequency of tooth loss due to exodontias (39%), which highlights the need for early diagnosis and intervention in these patients. Because 47% of the patients with AI present caries, exodontias can be justified. The high frequency of caries is consistent with previous reports in which more caries were observed in patients with AI than in healthy individuals.^14^ ^,^ ^22^ ^,^ ^23^ However, the number of teeth with caries in patients with AI in this study was similar to that found in healthy 12-year-old Chilean children^24^. This suggests that caries in individuals with AI could be explained by the time required to find a specialist that can diagnose them and provide adequate treatment, in addition to the intrinsic susceptibility of their defective enamel.

The median number of restorations was 3 (range of 0-26), which is higher than that reported in healthy 12-year-old Chilean patients who presented a frequency of 1.03 teeth restored.^24^ However, a study in healthy elderly Chilean adults observed an average of 8.9 restorations *per* individual,^25^ which indicated that our patients with AI, although much younger, often had a concerning number of restorations that was closer to the number seen in elderly adults than to that in young people in the same age range. On the other hand, 32% of the patients with AI presented periodontal pathology without differences among the different clinical subtypes, which is supported by the literature.^3^ ^,^ ^14^ This is probably because enamel alterations favor the retention of bacterial plaque. In addition, dental hypersensitivity *per se* makes adequate oral hygiene difficult.

Several studies have indicated the association of AI with malocclusions such as anterior open bite (AOB).^26^ ^-^ ^28^ Other reports of cases with different types of AI describe the presence of prognathism, posterior cross-bite and others anomalies.^11^ ^,^ ^27^ ^,^ ^29^ In the present study, malocclusions were observed in 43% of the individuals with AI without a significant difference among the different types. Eruption delay and agenesis were rare in this study, but low/high lip frenum insertion and ogival palate, which are not often reported in the literature, were observed in more than 20% of the individuals with AI.

Patients in this sample had a large number of teeth damaged by AI, with a median of 22 severely affected teeth (range of 4-31) at the time of the examination. It is interesting to note that all individuals with hypocalcified AI, alone or associated with hypoplastic AI, presented enamel with opaque gloss, rough texture and reduced thickness. The majority of patients with hypomature and hypoplastic AI phenotypes presented whitish enamel. The decrease in thickness was frequently observed in the hypoplastic type of AI, but a normal enamel thickness was present in 90% of the patients with hypomature AI and 60% in the combined type of hypomature/hypoplastic AI.

Finally, regarding clinical analysis, it is important to point out that, as reported in the literature,^6^ ^,^ ^10^ ^,^ ^15^ ^,^ ^17^ we also found great variability in the phenotypic expression of AI. We observed differences in the teeth of the same patient and between members of the same family.

### Radiographic analysis

In the radiographic evaluation, a similar frequency of patients with normal and diminished enamel thickness was observed. However, a decrease in the radiopacity of the enamel when compared with dentin was an important feature for the majority of the patients with AI. Patients with hypoplastic AI showed more frequent decreased enamel thickness and radiopacity. Most of the patients with hypomature AI presented with enamel with normal thickness and diminished radiopacity compared to dentin, and individuals with hypocalcified AI showed a tendency to have decreased enamel thickness and radiopacity. Although a 40% prevalence of taurodontism has been described in patients with AI,^6^ ^,^ ^13^ ^,^ ^30^ in the present study, a low percentage of individuals with this characteristic was found.

### Genealogical analysis

In this study, 23 families (56%) presented significantly more autosomal recessive inheritance patterns, which corroborates with Wright's study^10^ (2011), in which 50% of their families showed this type of inheritance. However, Chan^17^ (2011) reported 15% autosomal recessive inheritance, and Backman^15^ (1988) reported 12% of the cases as showing this type of inheritance. These differences may be due to the genetic background, the degree of consanguinity and differences in the allelic frequencies of the genes involved in the genetic etiology of the AI in the distinct population groups analyzed. With regard to the different clinical subtypes of AI with recessive inheritance patterns, it should be noted that, although the majority had HMAI (14 cases), this type of inheritance was also found in the clinical subtypes HC, HM/HP and HC/HP of AI. In addition, as previously mentioned, autosomal recessive inheritance was found in families with hypocalcified AI. These results contrast with the study by Wright^10^ (2011), which reported a high prevalence of hypomature AI with autosomal recessive inheritance and hypocalcified AI mainly associated with autosomal dominant inheritance. This could be due to the misinterpretation of incomplete information on pedigrees, given the impossibility of conducting clinical examinations for several members of the studied families. On the other hand, 37% of the families showed autosomal dominant inheritance patterns as described in studies involving several families.^15^ ^,^ ^17^

In our study, the X-linked pattern of inheritance was absent. However, a classic article on AI that involved a significant number of families established that approximately 5-10% of all the cases of AI are X-linked.^15^ In this context, Wright's work with 71 families reported that 6 (23%) cases presented X-linked patterns of inheritance. The study by Chan^17^ (2011), carried out with 39 families, showed that 4 of them (12%) presented this type of inheritance. Considering this information, in two of our families with hypomature/hypoplastic AI, there were two possible inheritance patterns involved, one of which was probably X-linked, but this was not possible to determine. This is due to a lack of information in the pedigree and to the fact that in these cases, it was difficult to determine the status of the condition in key individuals, given the number of restorations and missing teeth.

For these last few years, the heterogeneous genetic etiology of AI has been elucidated to a great extent, but not completely to date. Mutations in the genes *ENAM*, *COL17A1*, *LAMB3*, *ACPT*, *AMBN* and *IGTB6* are responsible for the hypoplastic forms of AI, with autosomal dominant and/or recessive inheritance patterns.^5^ ^,^ ^8^ On the other hand, hypomature forms of AI are caused by mutations in the genes *AMTN*, *KLK4*, *MMP20*, *WDR72*, *SLC24A4*, *C4orf26* and *GPR8*, all of which are associated with autosomal recessive inheritance.^5^ ^,^ ^8^ Finally, the only causative gene of hypocalcified autosomal dominant AI known thus far is *FAM83H*.^5^ ^,^ ^8^ For this reason, the genetic molecular analysis of a significant number of families should consider these 16 genes (and those involved in syndromic forms of AI) in the design of a targeted gene panel for massive sequencing for diagnostic purposes.

Although the main strength of this 13-years-long study is the inclusion of a large number of patients with AI in its sampling, it also has some limitations related to our nonrandom convenience sample formed by patients who came for treatment or were referred by other dentists due to severe alterations. Moreover, in many cases it was not possible to obtain complete information for the genealogical analysis, and the molecular genetic analyses are only recently underway.

## Conclusions

Considering the low worldwide prevalence of this pathology, i.e., 1 in 14,000 cases of all types of AI combined,^6^ this study involving a large group of affected families is the first to determine the frequency of clinical, radiographic and genealogical characteristics of five clinical subtypes of AI found in the Chilean population. Our study shows that autosomal recessive hypomature AI, autosomal dominant hypoplastic AI and autosomal recessive hypomature/hypoplastic AI are the most prevalent in this group and that the most frequently found characteristics are the presence of opaque white spots, reduction of thickness and enamel wear, malocclusions, and the presence of restorations, among others.

This work represents a source of complementary information for other studies and is of relevance and clinical utility for general dentists and specialists because it gathers information collected through the analysis of a significant number of patients with AI. Additionally, it provides more tools for the adequate diagnosis of this pathology, which will allow for early intervention by the professional and adequate preventive and restorative actions in a multidisciplinary framework.
